# Immune and senescence profiles associated with non-AIDS-defining cancer risk in people with HIV: a case-cohort study

**DOI:** 10.3389/fimmu.2025.1707510

**Published:** 2025-10-21

**Authors:** Carlos Pita-Martínez, María Ángeles Jiménez-Sousa, Javier Martinez-Picado, Carmen Elena Gómez Rodríguez, Carmen Fariñas, Pepa Galindo, Cristina Roca-Oporto, Jesús Santos, Paula Muñoz-García, Marta Rava, Salvador Resino, Rubén Martín-Escolano

**Affiliations:** ^1^ Unidad de Infección Viral e Inmunidad, Centro Nacional de Microbiología (CNM), Instituto de Salud Carlos III (ISCIII), Majadahonda, Madrid, Spain; ^2^ Centro de Investigación Biomédica en Red en Enfermedades Infecciosas (CIBERINFEC), Instituto de Salud Carlos III, Madrid, Spain; ^3^ IrsiCaixa, Badalona, Spain; ^4^ Germans Trias i Pujol Research Institute, Badalona, Spain; ^5^ Department of Infectious Diseases and Immunity, University of Vic-Central University of Catalonia, Vic, Spain; ^6^ Catalan Institution for Research and Advanced Studies, Barcelona, Spain; ^7^ Department of Molecular and Cellular Biology, Centro Nacional de Biotecnología, Consejo Superior de Investigaciones Científicas, Madrid, Spain; ^8^ Servicio de Enfermedades Infecciosas, Hospital Universitario Marqués de Valdecilla-IDIVAL, Santander, Spain; ^9^ Hospital Clínico Universitario de Valencia, Valencia, Spain; ^10^ Infectious Diseases, Microbiology, and Parasitology, Institute of Biomedicine of Seville/Virgen del Rocio University Hospital/CSIC/University of Seville, Seville, Spain; ^11^ Unidad de Enfermedades Infecciosas, Hospital Universitario Virgen de la Victoria, IBIMA, Málaga, Spain; ^12^ Centro Nacional de Epidemiología (CNE), Instituto de Salud Carlos III (ISCIII), Madrid, Spain

**Keywords:** NADCs, PWH, immune activation, senescence, biomarkers, gender differences

## Abstract

**Introduction:**

People with HIV (PWH) on effective antiretroviral therapy (ART) have an increased risk of developing Non-AIDS Defining Cancers (NADCs) compared to the general population, partly due to chronic inflammation and immune dysregulation. This study aimed to identify plasma biomarkers associated with the risk of developing NADCs in a cohort of PWH on ART.

**Methods:**

A case-cohort study was conducted within the Spanish CoRIS cohort, including 316 PWH on ART (71 cases and 245-individuals subcohort). Plasma levels of 24 immune regulation and senescence-associated secretory phenotype (SASP) biomarkers were quantified using Luminex technology. Cox proportional hazards regression models with Borgan II weights were used to assess the association between biomarker levels and the risk of NADC development (hazard ratios), adjusting for confounders. Effect modification by gender was also evaluated.

**Results:**

Higher baseline plasma levels of twelve biomarkers were significantly associated with increased NADC risk. The strongest associations were found for PD–L2 (aHR=3.33), PAI–1 (aHR=2.27), and MMP–1 (aHR=2.32). However, a distinct, gender-specific pattern was observed, with significant interactions found for nine biomarkers. Most interactions indicated a higher NADC risk increase in females, with the exception of CD80, TNF–β and IP–10, which indicated a relatively lower risk in females compared to males.

**Discussion:**

Plasma biomarkers of immune regulation and SASP are associated with NADC risk in PWH on long-term ART, highlighting the importance of gender-specific pathways in NADC development among PWH. Understanding these distinct profiles may guide future strategies for risk stratification, early detection, and personalized preventive care.

## 1 Introduction

The implementation of combination antiretroviral therapy (ART) has successfully transformed human immunodeficiency virus (HIV) infection into a chronic, manageable condition, leading to a substantial increase in the life expectancy of people with HIV (PWH) (1). This increased longevity, however, has been accompanied by a significant epidemiological shift in morbidity and mortality, moving from AIDS-defining illnesses to a spectrum of age-related diseases. Among these, non-AIDS-defining cancers (NADCs) have emerged as a primary cause of death in this population (2), representing a major clinical challenge in the current era of HIV care.

The incidence of numerous NADCs is significantly higher in PWH compared to the general population (3). This persistent and significant excess risk is not fully explained by the higher prevalence of traditional cancer risk factors such as smoking, alcohol consumption, or coinfections with oncogenic viruses like hepatitis B virus (HBV) and hepatitis C virus (HCV) (4). This disparity highlights the role of HIV-specific pathogenic mechanisms, particularly those related to inflammation and immunosenescence, that directly contribute to the rising incidence of NADCs (5), even among PWH who have achieved long-term viral suppression with ART (6). A deeper understanding of these underlying biological drivers is therefore critical for developing effective strategies for cancer prevention and control in PWH.

A central hypothesis to explain this heightened NADCs susceptibility implicates a state of chronic, low-grade inflammation that persists despite effective virological control (7). This phenomenon, often referred to as “inflammaging,” is multifactorial in origin, driven by factors including the persistence of HIV in latent tissue reservoirs, ongoing microbial translocation from a compromised gut mucosal barrier, the proinflammatory effects of co-pathogens, and irreversible damage to lymphoid tissue architecture (8). This systemic inflammatory state is biochemically reflected by elevated plasma levels of numerous inflammatory biomarkers, which have also been linked to increased all-cause mortality in PWH (9). Concurrently with chronic inflammation, PWH exhibit features of immune dysregulation and premature immunosenescence. A key aspect of this is T-cell exhaustion, a state of cellular dysfunction characterized by the sustained upregulation of inhibitory checkpoint receptors on the surface of T-lymphocytes (10). These include programmed cell death-1 (PD-1) and its ligands, as well as other co-inhibitory molecules like T-cell immunoglobulin and mucin-domain containing-3 (TIM-3) (11), among others. Therefore, comprehensively profiling plasma biomarkers that reflect these interconnected processes is essential to delineate better the pathways leading to NADCs in PWH and to identify robust signatures that could enhance clinical risk assessment and inform preventive strategies.

Additionally, a critical and under-investigated dimension of NADC pathogenesis in PWH is the role of gender-based biological differences. Women with HIV are known to exhibit higher T-cell activation and inflammation than men for a given viral load (12), and these immunological distinctions may create different risk profiles for chronic diseases (13). Understanding whether gender modifies the association between these biomarkers and NADC risk is therefore essential for developing personalized risk stratification strategies.

This study aimed to address these knowledge gaps by investigating the association between a broad panel of plasma biomarkers, representing key pathways of inflammation, immune regulation, and cellular senescence, and the risk of incident NADCs in a large, well-characterized cohort of PWH on long-term suppressive ART. A key focus of our study was to test whether these associations are modified by gender, in order to uncover distinct, gender-specific pathophysiological pathways that contribute to cancer risk in PWH.

## 2 Materials and methods

### 2.1 Study design and participants

We designed a case–cohort study nested within the CoRIS Cohort, a prospective, open, multicenter cohort of PWH ART-naïve at study entry. Data are standardized and structured following the HIV Cohorts Data Exchange Protocol (HICDEP) (14) and are subjected to rigorous internal quality control procedures on an annual basis. Participants are periodically monitored in line with standard clinical practice (15).

The study population was selected from 19,352 participants enrolled in CoRIS as of November 30, 2022. The inclusion criteria were: i) adults (≥18 years) on ART, ii) with at least one plasma sample stored in the HIV biobank 48 weeks after initiating ART (defined as the baseline sample), iii) having a suppressed HIV viral load (<50 copies/mL) at the time of the baseline sample. From participants fulfilling these criteria, cases were defined as those who developed an incident NADC at least three months after their baseline date. Individuals with non-melanoma skin cancers, metastasis or a prior history of the same type of NADC were excluded. The subcohort comprised a random sample of the entire source population.

The follow-up period for each individual began three months after the baseline sample date. The end of follow-up was defined as the date of the NADC diagnosis, death, last study visit, or administrative censoring date (November 30, 2022), whichever was first. After applying all criteria, the final study population consisted of 316 PWH, comprising 71 incident NADC cases and a subcohort of 245 individuals.

### 2.2 Clinical data and samples

Demographic, clinical, and virological data were sourced from the CoRIS database. This included demographics (age, gender, region of origin, education), HIV-related variables (transmission route, previous AIDS diagnosis, nadir and baseline CD4+ T-cell counts), and relevant comorbidities and lifestyle factors (smoking status, HCV and HBV coinfection), among others. Plasma samples were collected and processed at the baseline sample date and stored at -80 °C at the Spanish HIV BioBank until use.

### 2.3 Multiplex immunoassays

Plasma levels of 24 immune regulation and senescence-associated secretory phenotype (SASP) biomarkers were measured using a Luminex 200™ analyzer (Luminex Corporation, Austin, TX, United States) according to the manufacturer’s instructions. The panel included: cluster of differentiation (CD)28, CD36, CD80, epidermal growth factor (EGF), fibroblast growth factor 2 (FGF–2), growth differentiation factor 15 (GDF–15), hepatocyte growth factor (HGF), interleukin 1-alpha (IL–1α), IL–8, interferon gamma-induced protein 10 (IP–10), lymphocyte-activation gene 3 (LAG–3), monocyte chemoattractant protein 1 (MCP-1), MCP-2, metalloproteinase 1 (MMP–1), plasminogen activator inhibitor 1 (PAI–1), PD–1, programmed death-ligand 1 (PD–L1), PD–L2, stromal cell-derived factor 1-alpha (SDF–1α), TIM–3, tumor necrosis factor beta (TNF–β), tumor necrosis factor receptor I (TNF–RI), TNF–RII, and vascular endothelial growth factor A (VEGF–A). The measured raw fluorescence intensity (FI) values (arbitrary units, a.u.) were used as previously described (16).

### 2.4 Statistical analysis

For descriptive analysis, continuous variables were shown as median (interquartile range, IQR) and compared using the Mann-Whitney U test. Categorical variables were shown as absolute count (percentage), and compared using Chi-square or Fisher’s exact test, as appropriate.

To compare baseline biomarker levels between individuals who developed NADCs and those who did not, median with interquartile range (IQR) were calculated and compared using the Mann-Whitney U test. Additionally, to assess the cross-sectional association of baseline biomarker levels with NADC status, Generalized Linear Models (GLM) with a gamma distribution (log-link) were performed. These models were used to calculate both unadjusted and adjusted arithmetic mean ratios (AMR and aAMR, respectively). In addition, to assess the correlations among the 24 plasma biomarkers, pairwise Spearman’s rank correlation tests were performed.

Borgan II-weighted cause-specific Cox proportional hazards regression models (17) using robust standard errors (18) were used to estimate hazard ratios (HRs) and 95% confidence intervals (95% CIs) for the association between biomarker levels and the risk of NADC occurrence over time, with age as the time scale, and death as a competing event. The biomarker levels were first winsorized to minimize the influence of extreme outliers and then analyzed as continuous (log2-transformed) variables. Multivariable models were adjusted for demographic, clinical, and viro-immunological confounders, including: gender (male, female), region of origin (Spain, Europe (excluding Spain), Latin America, other/unknown), education (none or primary, secondary, university, other/unknown), transmission route (heterosexual, men who have sex with men (MSM), injecting drug users, other/unknown), smoking (never smoker, ex-smoker, smoker, unknown), prior AIDS diagnosis (yes, no), CD4+ T cell/mm^3^ (≥500, 350–499, <350, unknown), HCV (yes, no), and HBV coinfection (yes, no, unknown). The final adjusted model simultaneously included all these covariates, which were selected *a priori* based on their clinical relevance and not through a data-driven selection process.

To assess whether the associations between biomarker levels and the risk of NADC occurrence over time differed by gender, an interaction term was included between each biomarker and gender in both unadjusted and adjusted models. P-values for interaction were obtained using a likelihood ratio test (LRT) comparing a model with interaction with a nested model without interaction.

P-values were corrected for multiple testing using the False Discovery Rate (FDR) method (q-values) (19). A p-value <0.050 was considered statistically significant. A q-value <0.200 was used to identify potentially relevant associations. This less stringent q-value threshold is often employed in exploratory or hypothesis-generating studies to effectively balance the risk of false positives while minimizing the chance of missing true biological signals, particularly when the number of events is limited.

All statistical analyses were performed using the R statistical package (R version 4.3.1. R Foundation for Statistical Computing, Vienna, Austria).

## 3 Results

### 3.1 Individuals’ characteristics

The baseline characteristics of 316 PWH are detailed in **Table 1**. Overall, the cohort was predominantly male (84.8%), mostly from Spain (62.3%), with a median age of 41 years. Sexual transmission was the most frequent route, primarily among MSM (61.1%), followed by heterosexual transmission (29.1%). A prior AIDS diagnosis was present in 16.1% of participants, and the median baseline CD4+ T cell count was 614 cells/mm³. The most common antiretroviral regimen was based on nucleoside reverse transcriptase inhibitors (NRTI) combined with non-nucleoside reverse transcriptase inhibitors (NNRTI) (34.5%), followed by a combination of NRTI plus integrase inhibitors (II) (30.1%).

**Table 1:** Clinical, epidemiological, and virological profiles at baseline of people with HIV (PWH) on antiretroviral treatment stratified by the development of non-AIDS-defining cancers (NADCs).

	All patients	NADCs	Not NADCs	p-value
No.	316	71 (22.5%)	245 (77.5%)	
Age (years)	41 (33–49)	50 (44–60)	39 (32–45)	**<0.001**
Gender (male)	268 (84.8%)	56 (78.9%)	212 (86.5%)	0.163
Region				**0.033**
Spain	197 (62.3%)	51 (71.8%)	146 (59.6%)	
Europe (excluding Spain)	38 (12.0%)	11 (15.5%)	27 (11.0%)	
Latin America	64 (20.3%)	6 (8.5%)	58 (23.7%)	
Other/Unknown	17 (5.4%)	3 (4.2%)	14 (5.7%)	
Education				**0.002**
None or primary	55 (17.4%)	22 (31.0%)	33 (13.5%)	
Secondary	147 (46.5%)	33 (46.5%)	114 (46.5%)	
University	89 (28.2%)	12 (16.9%)	77 (31.4%)	
Other/Unknown	25 (7.9%)	4 (5.6%)	21 (8.6%)	
Smoking				**<0.001**
Never smoker	91 (28.8%)	5 (7.0%)	86 (35.1%)	
Ex-smoker	26 (8.2%)	10 (14.1%)	16 (6.5%)	
Smoker	90 (28.5%)	24 (33.8%)	66 (26.9%)	
Unknown	109 (34.5%)	32 (45.1%)	77 (31.4%)	
Transmission				**<0.001**
Heterosexual	92 (29.1%)	31 (43.7%)	61 (24.9%)	
MSM	193 (61.1%)	28 (39.4%)	165 (67.3%)	
IDU	16 (5.1%)	8 (11.3%)	8 (3.3%)	
Other/Unknown	15 (4.7%)	4 (5.6%)	11 (4.5%)	
HCV coinfection	24 (7.6%)	12 (16.9%)	12 (4.9%)	**0.002**
HBV coinfection (n = 286)	103 (36.0%)	39 (56.5%)	64 (29.5%)	**<0.001**
HIV markers				
Previous AIDS	51 (16.1%)	20 (28.2%)	31 (12.7%)	**0.003**
Nadir CD4+/mm3	289 (145–405)	203 (73–301)	317 (166–434)	**<0.001**
Baseline CD4+ T-cells/mm3 (n = 296)	614 (423–842)	528 (373–726)	631 (446–858)	**0.015**
Baseline CD4+/mm3				0.093
<350	50 (15.8%)	13 (18.3%)	37 (15.1%)	
350–499	56 (17.7%)	19 (26.8%)	37 (15.1%)	
≥500	205 (64.9%)	38 (53.5%)	167 (68.2%)	
Unknown	5 (1.6%)	1 (1.4%)	4 (1.6%)	
HIV antiretroviral therapy				0.030
NRTI + II	95 (30.1%)	13 (18.3%)	82 (33.5%)	
NRTI + PI	60 (19.0%)	21 (29.6%)	39 (15.9%)	
NRTI + NNRTI	109 (34.5%)	27 (38.0%)	82 (33.5%)	
Other	43 (13.6%)	9 (12.7%)	34 (13.9%)	
Unknown	9 (2.8%)	1 (1.4%)	8 (3.3%)	

Statistics: Categorical variables are reported as frequencies (percentages), while continuous variables are presented as medians (interquartile range).1 P-values were obtained through the Chisquare/Fisher test and the Mann-Whitney U test. Statistically significant differences are shown in bold. MSM, men who have sex with men; IDU, injecting drug users; AIDS, acquired immune deficiency syndrome; NRTI, nucleoside analogue HIV reverse transcriptase inhibitor; II, HIV integrase inhibitor; PI, HIV protease inhibitor; NNRTI, non-nucleoside analogue HIV reverse transcriptase inhibitor.

Significant differences were observed between PWH who developed NADCs and those who did not (**Table 1**). Individuals who developed NADCs were older, more frequently reported a history of heterosexual transmission, had a higher rate of smoking, lower nadir and baseline CD4+ T cell counts, and a higher frequency of prior AIDS diagnoses, as well as HCV and HBV coinfections. Differences were also observed for ART regimens, with NNRTI-based therapies being most represented in the NADC group (38%). The distribution of specific NADCs diagnosed in the NADC group is detailed in the **Supplementary Material** (**Supplementary Table S1**).

### 3.2 Biomarker levels and correlations

The baseline plasma levels for the 24 biomarkers in both NADC cases and the subcohort are detailed in **Supplementary Table S2**. While a trend towards higher median levels was observed for several biomarkers in the group that subsequently developed an NADC, formal statistical comparisons of these baseline levels did not yield statistically significant differences. This finding underscores that the association between these biomarkers and cancer development is more robustly captured through longitudinal risk modeling over time, as presented in Cox regression analyses, rather than through simple cross-sectional comparisons at a single time point.

The correlation matrix between the biomarkers is shown in **Supplementary Figure S1**. Notably, all pairwise correlations displayed were statistically significant (p <0.05) and revealed a landscape of predominantly moderate to strong positive correlations.

### 3.3 Plasma biomarkers associated with incident NADCs

**Figure 1:**
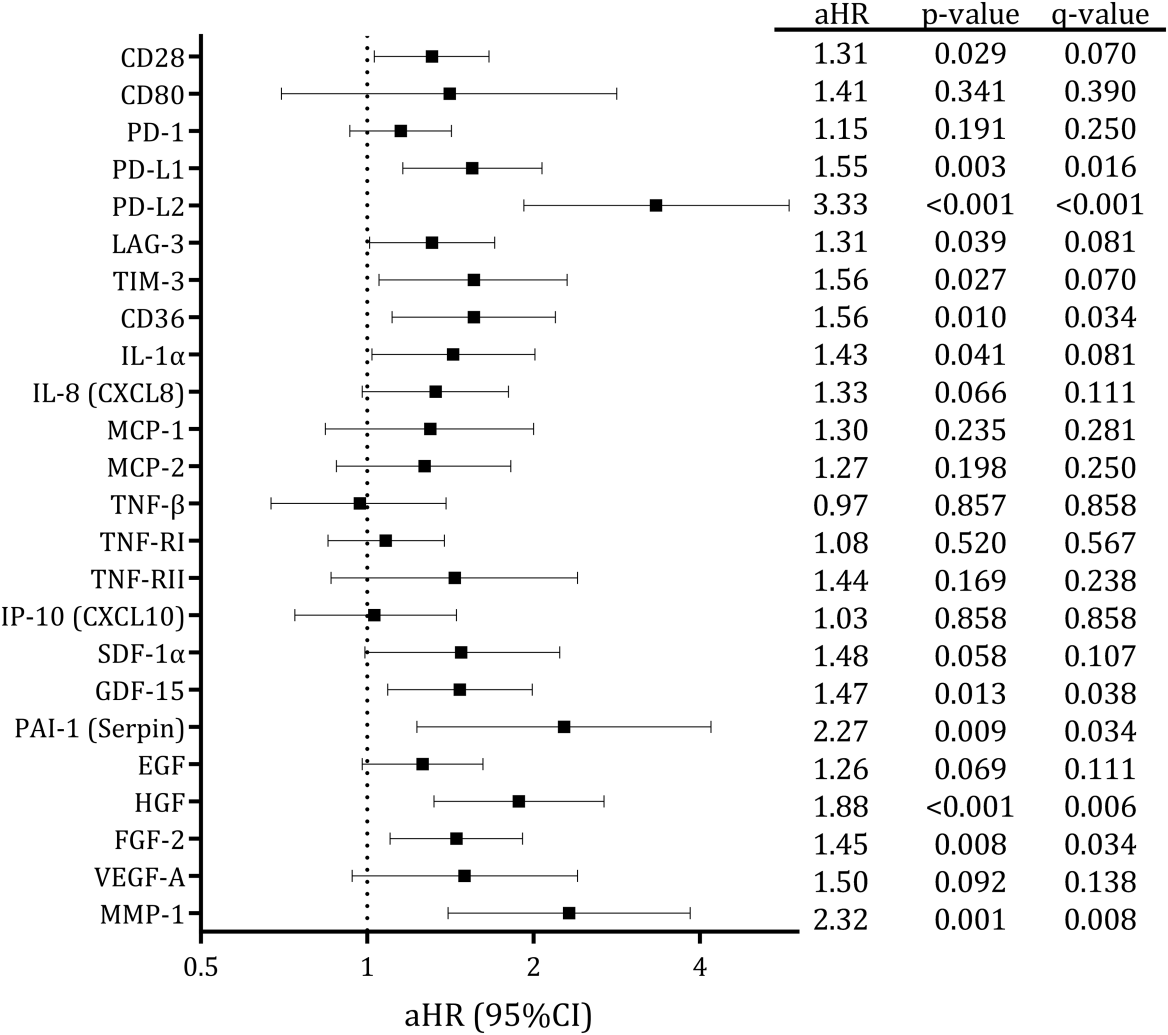
Adjusted associations of plasma markers with the development of NADCs in PWH on ART. Statistics: aHR and 95%CI were estimated with cause–specific Cox proportional hazards regression models with Borgan II weights. with age as time scale. Models were adjusted for relevant confounders (see Methods Section). The q–values represent p–values corrected for multiple testing using the False Discovery Rate (FDR). NADCs, non–AIDS–defining cancers; PWH, people with HIV; ART, antiretroviral treatment; aHR, adjusted Hazard Ratio; 95%CI, 95% of confidence interval; p, level of significance; q, corrected level of significance; CD, cluster of differentiation; EGF, epidermal growth factor; FGF, fibroblast growth factor; GDF, growth differentiation factor; HGF, hepatocyte growth factor; IL, interleukin; IP, interferon gamma–induced protein; LAG, lymphocyte activation gene; MCP, monocyte chemoattractant protein; MMP, matrix metalloproteinase; PAI, Plasminogen Activator Inhibitor; PD–1, programmed cell death protein; PD–L, programmed death–ligand; SDF, stromal cell–derived factor; TIM, T–cell immunoglobulin and mucin domain; TNF, tumour necrosis factor; TNF–R, tumour necrosis factor receptor; VEGF, vascular endothelial growth factor.

The associations between baseline plasma biomarkers and the NADC risk are presented in **Supplementary Table S3**. In the adjusted models (**Figure 1**), higher baseline levels of twelve biomarkers were significantly associated with an increased risk of incident NADC: CD28 (adjusted HR (aHR) (95% CI)=1.31 (1.03 – 1.66), q=0.070), PD–L1 (aHR (95% CI)=1.55 (1.16 – 2.07), q=0.016), PD–L2 (aHR (95% CI)=3.33 (1.92 – 5.80), q<0.001), LAG–3 (aHR (95% CI)=1.31 (1.01 – 1.70), q=0.081), TIM–3 (aHR (95% CI)=1.56 (1.05 – 2.30), q=0.070), CD36 (aHR (95% CI)=1.56 (1.11 – 2.19), q=0.034), IL–1α (aHR (95% CI)=1.43 (1.02 - 2.01), q=0.081), GDF1–5 (aHR (95% CI)=1.47 (1.09 - 1.99), q=0.038), PAI–1 (aHR (95% CI)=2.27 (1.23 - 4.19), q=0.034), HGF (aHR (95% CI)=1.88 (1.32 - 2.68), q=0.006), FGF–2 (aHR (95% CI)=1.45 (1.10 - 1.91), q=0.034), and MMP–1 (aHR (95% CI)=2.32 (1.40 - 3.84), q=0.008). Furthermore, a similar trend was observed for IL–8 (aHR (95% CI)=1.33 (0.98 - 1.80), q=0.111), SDF–1α (aHR (95% CI)=1.48 (0.99 - 2.23), q=0.107), and EGF (aHR (95% CI)=1.26 (0.98 - 1.62), q=0.111), which were borderline significant.

### 3.4 Gender as an effect modifier of biomarker association with NADC risk

Before assessing gender as an effect modifier on NADC risk over time, we first examined baseline differences in biomarker levels between males and females within both the NADC case group and the non-NADC subcohort (**Supplementary Table S4**). No significant differences were found among the NADC and non-NADC groups, except for MCP-1 (p=0.008) and HGF (p=0.032) within the subcohort.

We found that gender modified the association between several baseline plasma biomarkers and the risk of developing NADC (unadjusted models are shown in **Supplementary Table S5**). In the adjusted models (**Table 2**), we observed statistically significant interactions for CD80 (q=0.047), PD–L2 (q=0.077), MCP–1 (q=0.006), MCP–2 (q=0.125), TNF–β (q=0.125), IP–10 (q<0.001), EGF (q=0.125), HGF (q=0.006), and MMP–1 (q=0.022). For the majority of these (PD–L2, MCP–1, MCP–2, EGF, HGF, and MMP–1), the interactions indicated that higher biomarker levels conferred a stronger NADC risk in females than in males. However, the effect estimates for several of these markers in females (PD–L2, MCP–1, MCP–2, HGF, and MMP–1) should be interpreted with caution due to wide confidence intervals, which reflect the limited statistical power in this subgroup. Notably, CD80, TNF–β and IP–10 displayed the opposite effect, indicating that higher levels of this biomarker were associated with a relatively lower NADC risk in females compared to males.

**Table 2:** Adjusted association of plasma markers of immune regulation and senescence-associated secretory phenotype (SASP) at baseline with the development of non–AIDS–defining cancers (NADCs) during the follow–up time in people with HIV (PWH) on antiretroviral treatment, by gender.

Category	Marker	Female individuals	Male individuals	Interaction	
		aHR (95%CI)	aHR (95%CI)	p-value	q-value
Immune regulation	CD28	1.17 (0.31 – 4.36)	1.32 (1.03 – 1.69)	0.758	0.843
	CD80	0.12 (0.00 – 4.54)	1.67 (0.83 – 3.35)	**0.010**	**0.047**
	PD–1	1.26 (0.73 – 2.18)	1.12 (0.88 – 1.42)	0.605	0.806
	PD–L1	2.25 (1.18 – 4.31)	1.39 (0.95 – 2.02)	0.155	0.265
	PD–L2	14.40 (1.96 – 105.57)	2.77 (1.55 – 4.97)	**0.019**	**0.077**
	LAG–3	1.53 (0.68 – 3.46)	1.27 (0.94 – 1.71)	0.552	0.780
	TIM–3	2.61 (0.90 – 7.53)	1.39 (0.89 – 2.18)	0.151	0.265
Senescence–associated secretory phenotype (SASP)	CD36	1.56 (0.64 – 3.78)	1.56 (1.09 – 2.24)	0.999	0.999
	IL–1α	1.26 (0.28 – 5.73)	1.45 (1.00 – 2.11)	0.763	0.843
	IL–8 (CXCL8)	0.92 (0.20 – 4.22)	1.37 (1.01 – 2.11)	0.370	0.592
	MCP–1	4.07 (1.52 – 10.92)	0.90 (0.53 – 1.51)	**<0.001**	**0.006**
	MCP–2	3.02 (1.26 – 7.23)	1.14 (0.78 – 1.67)	**0.040**	**0.125**
	TNF–β	0.57 (0.22 – 1.44)	1.10 (0.75 – 1.59)	**0.045**	**0.125**
	TNF–RI	0.98 (0.40 – 2.41)	1.10 (0.86 – 1.41)	0.701	0.843
	TNF–RII	2.93 (0.89 – 9.61)	1.26 (0.74 – 2.14)	0.097	0.193
	IP–10 (CXCL10)	0.42 (0.19 – 0.92)	1.25 (0.92 – 1.71)	**<0.001**	**<0.001**
	SDF–1α	1.57 (0.57 – 4.32)	1.47 (0.94 – 2.30)	0.882	0.920
	GDF–15	1.88 (0.64 – 5.48)	1.43 (1.05 – 1.95)	0.512	0.769
	PAI–1 (Serpin)	2.70 (0.36 – 10.34)	2.17 (1.05 – 4.46)	0.772	0.843
	EGF	2.01 (1.07 – 3.77)	1.14 (0.87 – 1.50)	**0.047**	**0.125**
	HGF	6.31 (2.66 – 14.95)	1.41 (0.94 – 2.11)	**<0.001**	**0.006**
	FGF–2	2.32 (1.32 – 4.10)	1.35 (1.01 – 1.82)	0.062	0.135
	VEGF–A	0.54 (0.05 – 5.67)	1.69 (1.04 – 2.74)	0.059	0.135
	MMP–1	8.47 (2.98 – 24.07)	1.79 (1.05 – 3.07)	**0.004**	**0.022**

Statistics: Data were calculated by Cox proportional hazards regression models with Borgan weighting. Age was used as the time scale and death as a competing event in the models. Multivariable models were adjusted for relevant confounders (see Methods Section). The q–values represent p–values corrected for multiple testing using the False Discovery Rate (FDR). Statistically significant differences are shown in bold. aHR, adjusted HR; 95%CI, 95% of confidence interval; p, level of significance; q, corrected level of significance; CD, cluster of differentiation; EGF, epidermal growth factor; FGF, fibroblast growth factor; GDF, growth differentiation factor; HGF, hepatocyte growth factor; IL, interleukin; IP, interferon gamma–induced protein; LAG, lymphocyte activation gene; MCP, monocyte chemoattractant protein; MMP, matrix metalloproteinase; PAI, Plasminogen Activator Inhibitor; PD–1, programmed cell death protein; PD–L, programmed death–ligand; SDF, stromal cell–derived factor; TIM, T–cell immunoglobulin and mucin domain; TNF, tumour necrosis factor; TNF–R, tumour necrosis factor receptor; VEGF, vascular endothelial growth factor.

## 4 Discussion

This study identified several biomarkers associated with NADC risk in PWH on long-term ART, highlighting a significant effect modification by gender. Our central finding is that the magnitude and, in some cases, the direction of these associations differ substantially between females and males. This suggests that the biological pathways driving carcinogenesis in the context of chronic HIV infection are sexually dimorphic, a critical consideration for future research and clinical practice.

Despite effective virological suppression, PWH on ART remain in a state of inflammaging (7), characterized by a sustained increase in inflammatory cells and proinflammatory mediators. Our data visually support this, showing a widespread elevation of most biomarkers in PWH who subsequently developed an NADC. Furthermore, the strong positive correlations among these proteins point to a highly co-regulated, systemic pro-inflammatory state. In this context, we identified a broad profile of immune regulation and SASP biomarkers associated with an increased risk of NADCs. This included twelve biomarkers, including CD28, PD–L1, PD–L2, LAG–3, TIM–3, CD36, IL–1α, GDF–15, PAI–1, HGF, FGF–2, and MMP–1, and notably, a strong trend of association for three others (IL–8, SDF–1α, and EGF). These markers represent key pathways implicated in cancer pathogenesis. Elevated levels of immune checkpoint proteins such as PD–L1, PD–L2, LAG–3, and TIM–3, alongside the co-stimulatory molecule CD28, point towards a state of dysregulated T-cell and immune activation and exhaustion, potentially contributing to a dysfunctional, exhausted, and inflammatory immune phenotype that compromises the host’s ability for an effective anti-tumor response (10, 20, 21). Similarly, the association with IL–1α, GDF–15 and PAI–1, key components of SASP, highlights accelerated immune ageing, which is conducive to tumorigenesis (22–24). Concurrently, markers like HGF, EGF, MMP–1 and the scavenger receptor CD36 reflect processes of chronic inflammation, tissue remodeling, and metabolic reprogramming, which facilitate cancer cell proliferation and survival (25–27). Finally, increased FGF–2 levels, further supported by trends in SDF–1α and IL–8, imply activation of pro-angiogenic pathways, which are critical for supporting tumor growth through enhanced nutrient and oxygen delivery (28–30). Although these pathways are essential for maintaining self-tolerance and limiting immunopathology, their chronic activation in the context of HIV leads to a progressive loss of their functions. Collectively, these findings delineate a complex and multifactorial biological state that may predispose PWH to the development of NADCs.

Within this multifactorial biological state, three biomarkers stood out with the strongest associations to NADC risk: PD–L2, PAI–1, and MMP–1. The particularly strong signal from PD–L2 points to a profound state of T-cell exhaustion and immune evasion, a critical mechanism that allows nascent cancer cells to escape immune destruction (31). This is complemented by the high levels of PAI–1, a key SASP component that not only signifies accelerated cellular aging but also actively promotes a pro-tumorigenic microenvironment through its inflammatory and tissue-remodeling properties (24). Finally, the robust association with MMP–1 underscores the importance of active extracellular matrix degradation, a fundamental step for tumor cell invasion (32). The convergence of these three markers paints a clear picture of the most critical pathogenic pathways at play: compromised anti-tumor immunity, a senescence-driven inflammatory milieu, and the enzymatic machinery for tissue invasion, which together form a potent triad driving NADC development in this population.

Building on the general associations, our study’s most critical finding was the significant effect modification by gender. While previous studies have linked inflammation to NADC risk in PWH (5–7), and others have described immunological differences between genders (12, 13), our work provides the crucial link between these two observations, presenting novel evidence that these immunological differences translate into gender-specific biomarker signatures of NADC risk. This phenomenon is increasingly recognized in HIV-associated comorbidities (33), and aligns with growing evidence that chronic inflammation and immune activation pathways are regulated in a gender-specific manner (34). For several key biomarkers—including the immune checkpoint PD–L2, the proinflammatory chemokines MCP–1 and MCP–2, the growth factors EGF and HGF, and the tissue-remodeling enzyme MMP–1—our interaction tests confirmed that the pro-tumorigenic association is significantly stronger in females. It is crucial to note, however, that while the direction of this gender-specific effect was consistent, the precise magnitude of the risk in females should be interpreted with caution. The female-specific estimates for these markers were accompanied by wide confidence intervals, reflecting lower statistical power in this subgroup. Nevertheless, this multi-faceted profile suggests that in females with HIV, a combination of immune exhaustion, chronic inflammation, and aberrant cellular growth creates a highly pro-tumorigenic environment. This is biologically plausible, as females are known to mount stronger innate and adaptive immune responses, which, in the context of a chronic antigen stimulus like HIV, may lead to more pronounced and damaging chronic inflammation (33–35).

In contrast, CD80, TNF–β, and IP–10 showed a significant interaction in the opposite direction, with a relatively lower NADC risk in females compared to males at higher levels of these biomarkers. This observation may be explained by the complex and often dual roles these molecules play in tumor immunology. CD80 is a co-stimulatory molecule that can either activate anti-tumor T-cells or deliver inhibitory signals (36). TNF–β is a cytokine with both pro-apoptotic and pro-inflammatory, tumor-promoting capabilities (37). Similarly, IP–10 is a complex chemokine that can recruits anti-tumor T-cells and also promote inflammation and angiogenesis (38). The opposing interaction effect observed could reflect these context-dependent functions, potentially influenced by the distinct hormonal and immunological environments of each gender. This specific finding warrants further investigation to unravel the precise mechanisms behind these gender-specific interactions. These findings underscore that a universal biomarker approach is likely insufficient and that gender-specific strategies are essential for developing effective predictive tools for NADC risk in PWH, potentially involving distinct sets of markers for male and female individuals.

Our study has several limitations. First, the number of female participants, and particularly female cases, was small, which limits the statistical power to detect interaction effects, meaning some differences may not have reached statistical significance. Despite this, we identified several robust interactions, suggesting the impact we did detect is of considerable magnitude. Second, biomarker measurements were performed at a single time point, which does not capture the dynamic nature of inflammatory processes in PWH over time (39). Third, while we adjusted for a wide range of potential confounders, residual confounding from unmeasured factors, such as diet or body mass index, cannot be entirely ruled out because of incomplete data availability in CoRIS. Finally, our analysis grouped all NADCs together; future studies with larger numbers of events could explore whether these biomarker signatures are specific to certain cancer types.

Nevertheless, this study has several strengths. First, it is nested within a large, well-characterized national cohort (CoRIS) with long-term follow-up and rigorously collected data. Second, the case-cohort design is an efficient and statistically valid method for studying rare outcomes in large cohorts. Third, our study was limited to the analysis of soluble plasma biomarkers, which precludes an assessment of cellular-level contributions to the observed phenomena. Fourth, we analyzed a broad panel of 24 biomarkers, allowing for a comprehensive assessment of multiple biological pathways simultaneously.

Fifth, the use of age as the time scale in our Cox models is another key strength, given its strong association with cancer risk, as using time-on-study may bias results (40). Finally, death was treated as a competing event to account for the possibility that individuals may die before developing an NADC, thereby precluding its occurrence and potentially biasing risk estimates (41).

In conclusion, plasma biomarker levels of immune regulation and SASP are associated with NADC risk in PWH on long-term ART. These associations are significantly modified by gender, with key pathways of inflammation, tissue remodeling, and cellular growth conferring a stronger pro-tumorigenic effect in females. These findings highlight the importance of considering gender-specific inflammatory and immunosenescence pathways in NADC development among PWH and pave the way for more personalized risk-stratification strategies.

## Data Availability

The raw data supporting the conclusions of this article will be made available by the authors, without undue reservation.

## Supplementary material

Appendix A CoRIS cohort.

Supplementary Table 1 Type and frequency of non-AIDS-defining cancers (NADCs) developed during the follow-up time in 71 people with HIV (PWH) on antiretroviral treatment.

Supplementary Table 2 Plasma levels of immune regulation and senescence-associated secretory phenotype (SASP) biomarkers at baseline in people with HIV (PWH) on antiretroviral treatment without and with non–AIDS–defining cancers (NADCs) during the follow–up time.

Supplementary Table 3 Association of plasma markers of immune regulation and senescence-associated secretory phenotype (SASP) at baseline with the development of non–AIDS–defining cancers (NADCs) during the follow–up time in people with HIV (PWH) on antiretroviral treatment.

Supplementary Table 4 Plasma levels of immune regulation and senescence-associated secretory phenotype (SASP) biomarkers at baseline in people with HIV (PWH) on antiretroviral treatment stratified by gender and NADC development.

Supplementary Table 5 Unadjusted association of plasma markers of immune regulation and senescence-associated secretory phenotype (SASP) at baseline with the development of non–AIDS–defining cancers (NADCs) during the follow–up time in people with HIV (PWH) on antiretroviral treatment, by gender.

Supplementary Figure 1 Correlation matrix of plasma levels of immune regulation and senescence-associated secretory phenotype (SASP) biomarkers at baseline in people with HIV (PWH) on antiretroviral treatment.
