# Supplementary material for: Immune and senescence profiles associated with non-AIDS-defining cancer risk in people with HIV: a case-cohort study
Source: Front Immunol. 2025 Oct 21;16:1707510. doi: 10.3389/fimmu.2025.1707510 (PMC12583951; doi:10.3389/fimmu.2025.1707510)
Supplement: Appendix A — CoRIS cohort. [file DataSheet1.docx]

Supplementary Material

# Appendix A. CoRIS cohort.

***CoRIS Executive committee:***

Santiago Moreno, Inma Jarrín, David Dalmau, M Luisa Navarro, Federico Garcia, Eva Poveda, Jose Antonio Iribarren, Félix Gutiérrez, Francesc Vidal, Juan Berenguer, Juan González.

***Centres and investigators involved in CoRIS cohort are listed below:***

**CoRIS Coordination Unit**

Inma Jarrín, Cristina Moreno, Marta Rava, Rebeca Izquierdo, Cristina Marco-Sánchez, Teresa Gómez-García.

**BioBanK HIV Hospital General Universitario Gregorio Marañón**

José Luis Jiménez.

**Hospital General Universitario Dr Balmis (Alicante)**

Sergio Reus, Irene Portilla, Esperanza Merino, Gema García, José Sánchez-Payá, Juan Carlos Rodríguez, Livia Giner, Joaquín Portilla, Vicente Boix, Diego Torrus, Julia Portilla-Tamarit, Héctor Pinargote.

**Hospital Universitario de Canarias (San Cristóbal de la Laguna)**

María Remedios Alemán, Nereyda Tosco-García, Ana López Lirola, Dácil García, Felicitas Díaz-Flores, María del Mar Alonso, Ricardo Pelazas, María Inmaculada Hernández, Lucia Romero-Acevedo, Abraham Bethencourt-Padilla, Daniel Rodríguez-Díaz, Ana María Godoy-Reyes.

**Hospital Universitario Central de Asturias (Oviedo)**

Víctor Asensi, Rebeca Cabo Magadan, Lorena Fernández, Javier Díaz-Arias

**Hospital Universitario 12 de Octubre (Madrid)**

Federico Pulido, Rafael Rubio, M Asunción Hernando, Otilia Bisbal, David Rial-Crestelo, María de Lagarde, Laura Bermejo, Mireia Santacreu, Juan Martín Torres, Belén Sánchez-López.

**Servicio de Enfermedades Infecciosas. Hospital Universitario Donostia. Instituto de Investigación BioDonostia (Donostia-San Sebastián)**

José Antonio Iribarren, Xabier Kortajarena, Claudia Nevado Pavón, Xabier Camino, Miguel Ángel Goenaga, M Jesús Bustinduy, Harkaitz Azkune, Maialen Ibarguren, Ignacio Álvarez-Rodriguez, Leire Gil-Alonso, Francisco Carmona-Torre, Ana Bayona Carlos, Maialen Lekuona Sanz.

**Hospital General Universitario De Elche (Elche)**

Félix Gutiérrez, Catalina Robledano, Mar Masiá, Sergio Padilla, Rafael Pascual, Marta Fernández, Antonio Galiana, José Alberto García, Xavier Barber, Javier García Abellán, Guillermo Telenti, Ángela Botella, Paula Mascarell, Lidia García-Sánchez, Nuria Ena, Leandro López, Jennifer Vallejo, Nieves Gonzalo-Jiménez, Montserrat Ruiz, Christian Ledesma, Santiago López, María Espinosa-Pérez, Ana Quiles, María del Mar Alcalde-Encinas, José García-García, Rosario Hernández-Ros, José Carlos Escribano, Marouane Menchi-Elanci, María del Mar García Navarro.

**Hospital General Universitario Gregorio Marañón (Madrid)**

Cristina Diez, Isabel Gutiérrez, Juan Berenguer, Margarita Ramírez, Teresa Aldamiz-Echevarría, Francisco Tejerina, Leire Pérez-Latorre, Chiara Fanciulli, Saray Corral.

**Hospital Universitari de Tarragona Joan XXIII (Tarragona)**

Joaquim Peraire, Anna Rull, Anna Martí, Consuelo Viladés, Beatriz Villar, Lluïsa Guillem, Silvia Chafino, Marina Flores, Francesc Vidal.

**Hospital Universitario y Politécnico de La Fe (Valencia)**

Marta Montero-Alonso, María Tasias-Pitarch, Eva Calabuig-Muñoz, Miguel Salavert-Lletí, Juan Fernández-Navarro, Rosa Blanes-Hernández, Jennifer Sánchez-Guevara.

**Hospital Universitario La Paz/IdiPAZ (Madrid)**

Juan González-García, José Ignacio Bernardino, Ana Delgado-Hierro, José Ramón Arribas, Víctor Arribas, Juan Miguel Castro, Luis Escosa, Iker Falces, Pedro Herranz-Pinto, Alicia González-Baeza, María Luz Martín-Carbonero, Rafael Micán, Rocío Montejano, María Luisa Montes, Luis Ramos-Ruperto, Berta Rodés Soldevila, Talia Sainz, Elena Sendagorta, Carmen Busca, Joanna Cano-Smith, Rosa de Miguel, María del Mar Arcos-Rueda, Alejandro de Gea-Grela, Nerea Iniesta-Arandia, Alejandro Díez-Vidal, María Jesús Roldán-Cabrales, Carlos Oñoro-López, Jara Llenas García, Laura Lucía Checa Daimiel.

**Hospital Universitari Mutua Terrassa (Terrassa)**

David Dalmau, Marina Martinez, Angels Jaén, Mireia Cairó, Javier Martinez-Lacasa, Roser Font, Laura Gisbert.

**Hospital Universitario de La Princesa (Madrid)**

Ignacio de los Santos, Alejandro de los Santos, Lucio García-Fraile, Enrique Martín-Gayo, Ildefonso Sánchez-Cerrillo, Ángela Gutiérrez, Carmen Sáez, Ana Barrios Blandino, Azucena Bautista, Marianela Ciudad, María Aguilera García, Violeta Sampériz Rubio, Javier Pérez Serrano, Isabel Belmonte Martín de Santa Olalla.

**Hospital Universitario Ramón y Cajal (Madrid)**

Santiago Moreno, Santos del Campo, José Luis Casado, Fernando Dronda, Ana Moreno, María Jesús Pérez-Elías, Sergio Serrano-Villar, María Jesús Vivancos-Gallego, Javier Martínez-Sanz, Alejandro Vallejo Tiller, Matilde Sánchez-Conde, José Antonio Pérez-Molina, José Manuel Hermida, Erick De La Torre Tarazona, Elena Moreno del Olmo, Laura Martín Pedraza, Claudio Díaz García, Jorge Díaz Álvarez, Alejandro García-García, Raquel Ron-González, Sergio Calderón Vicente, Roser Navarro Soler, Sara Saiz-Baggetto, Ana del Amo-de Palacios, Laura Luna, Miguel Antón Ámez Segovia, Sara Martín Colmenarejo, Cristina Chica.

**Hospital General Universitario Reina Sofía (Murcia)**

Enrique Bernal, María Dolores Hernández, Antonia Alcaraz, Joaquín Bravo, Ángeles Muñoz Pérez, Cristina Tomás Jimenez, Salvador Valero Cifuentes, Eva García-Villalba, Román González Hipólito, Elena Guijarro-Westermeyer, Rodrigo Martínez-Rodríguez, José Miguel Gómez Verdú.

**Hospital Universitario Clínico San Cecilio (Granada)**

Federico García, Clara Martínez, Maite Laperal, Leopoldo Muñoz Medina, Marta Álvarez-Estevez, Natalia Chueca-Porcuna, David Vinuesa-García, Adolfo de Salazar-González, Ana Fuentes-López, Emilio Guirao, Andrés Ruiz-Sancho, Francisco Anguita, Naya Faro, Lucia Chaves, Marta Illescas, Paloma Muñoz-Baez, Lucía Pérez, Ana Alberola Romano, Alberto Vazquez Blanquiño, Lucía Guillén-Zafra, Javier Martínez de Victoria-Carazo.

**Centro Sanitario Sandoval (Madrid)**

Jorge Del Romero-Guerrero, Montserrat Raposo, Teresa Puerta-López, Mar Vera, Juan Ballesteros, Begoña Baza, Laura Dans Villán, Ruben Linares Navarro, Ines Armenteros Yeguas, Eva Orviz-García, Santiago Fernández Castelao.

**Hospital Universitario Son Espases (Palma de Mallorca)**

Melchor Riera, Antonio Vanrell, María Peñaranda, Mª Angels Ribas, Antoni A. Campins, Mercedes Garcia-Gasalla, Francisco J Fanjul, Javier Murillas-Angoiti, Luisa Martin-Pena, Francisca Artigues, Sophia Pinecki, Adrián Ferre.

**Hospital Universitario Virgen de la Victoria (Málaga)**

Jesús Santos, María López-Jódar, Cristina Gómez-Ayerbe, Isabel Viciana, Rosario Palacios.

**Hospital Universitario Virgen del Rocío (Sevilla)**

Luis Fernando López-Cortés, Silvia Llaves-Flores, Inmaculada Rivas Jeremías, Nuria Espinosa, Cristina Roca-Oporto, Marta Herreros-Romero, César Sotomayor de la Piedra, Abraham Saborido Alconchel, Manuel Francisco Liroa, Jesús Fernández Plaza.

**Hospital Universitario de Bellvitge (Hospitalet de Llobregat)**

Juan Manuel Tiraboschi, Arkaitz Imaz, María Saumoy, Analuz Fernandez, Jaime Vega Costa, Daniel Medina Gamito.

**Hospital Costa del Sol (Marbella)**

Julián Olalla, Javier Pérez, Alfonso del Arco, Javier de la Torre, Francisca Ruiz.

**Hospital General Universitario Santa Lucía (Cartagena)**

Onofre Juan Martínez, Lorena Martinez, Francisco Jesús Vera, Josefina García, Begoña Alcaraz, Sergio Guillén Martínez, Patricia Carles García.

**Complejo Hospitalario Universitario a Coruña (CHUAC) (A Coruña)**

Álvaro Mena, Berta Pernas, Pilar Vázquez, Soledad López, Brais Castelo.

**Hospital Universitario Virgen de la Arrixaca (El Palmar)**

Carlos Galera, Marian Fernández, Helena Albendin, Antonia Castillo, Asunción Iborra, Antonio Moreno, M Angustias Merlos, Almudena Ortuño.

**Hospital Universitario Infanta Sofía (San Sebastián de los Reyes)**

Inés Suarez-García, Eduardo Malmierca, Patricia González-Ruano, M Pilar Ruiz, Luz Balsalobre, Ángela Somodevilla, Rebeca Fuerte Martínez.

**Hospital Clínico San Carlos (Madrid)**

Vicente Estrada, Nieves Sanz, Noemí Cabello-Clotet, María José Núñez, Ana Muñoz, Juncal Pérez-Somarriba, Reynaldo Homen, Rafael Rubio-Martín, Susana Olmedo, Julia Barrado.

**Hospital Universitario Príncipe de Asturias (Alcalá de Henares)**

José Sanz, Cristina Hernández-Gutiérrez, María Novella-Mena.

**Hospital Clínico Universitario de Valencia (Valencia)**

María José Galindo, Sandra Pérez Gómez, Ana Ferrer.

**Hospital Reina Sofía (Córdoba)**

Antonio Rivero-Román, Laura Ruiz-Torres, Antonio Rivero-Juárez, Pedro López-López, Mario Frias-Casas, Ángela Camacho, Ignacio Pérez, Diana Corona, Javier Manuel Caballero, Marina Gallo Marín, María Casares, Lucía Ríos-Muñoz, Claudia Ferreira-Tata, María Carrasquilla.

**Hospital Universitario Severo Ochoa (Leganés)**

Rafael Rodríguez-Rosado Martinez-Echevarría.

**Hospital Universitario Virgen de Valme (Sevilla)**

Juan Macías Sánchez, Pilar Rincón, Luis Miguel Real, Anaïs Corma, Jésica Martín-Carmona.

**Hospital Álvaro Cunqueiro (Vigo)**

Eva Poveda, Alexandre Pérez, Luis Morano, Celia Miralles, Antonio Ocampo, Jacobo Alonso, Inés Martínez, Aida López-López.

**Hospital Universitario Marqués de Valdecilla**

M Carmen Fariñas Álvarez, Claudia González Rico, Noelia Ruiz Alonso, Carlos Armiñanzas Castillo, Francisco Arnaiz de las Revillas Almajano, Manuel Gutiérrez Cuadra, Raúl Parra Fariñas, Paula Runza Buznego, Aitziber Illaro Uranga.

**Hospital Clínic de Barcelona**

José Luis Blanco Arévalo, Pilar Callau Cabrera, Josep Mallolas Masferrer, José Alcamí Pertejo, Sonsoles Sánchez-Palomino, Núria Climent, Ana González-Cordón, Montse Laguno Centeno, María Martínez-Rebollar, Juan Ambrosioni, Berta Torres, Lorena de la Mora, Alexy Inciarte, Esteban Martínez, José María Miró, Abiu Sempere, Julia Calvo, Iván Chivite, David García, Alberto Foncillas, Daniela Malano, Montse Plana, Roger Llobet, Estela Solbes, Octavi Roman, Rona Sagarra, Vanesa Guilera, Gemma Olmeda, Paula Arreba, María José Rodríguez, Emma Fernández, Ana Rodríguez, Alba ortega, Sergi Anguera, Raquel Aguiló, Laura Novell.

**Centro Nacional de Microbiología**

Luis Miguel Bedoya Del Olmo, Manuela Beltran Vicente, Mercedes Bermejo Herrero, Maria Esther Calonge Errejon, Laura Capa Muñoz, Maria Teresa Coiras Lopez, Francisco Diez Fuertes, Javier Garcia Perez, Nuria Gonzalez Fernandez, Elena Mateos De Las Moreras, Maria Teresa Perez Olmeda, Victor Sanchez Merino, Maria Eloisa Yuste Herranz.

# Supplementary Table 1. Type and frequency of non-AIDS-defining cancers (NADCs) developed during the follow-up time in 71 people with HIV (PWH) on antiretroviral treatment.

| **Type of NADC** | **Frequency** |
| --- | --- |
| Lung cancer | 19 |
| Hodgkin's lymphoma | 8 |
| Prostate cancer | 7 |
| Colon cancer | 4 |
| Laryngeal cancer | 4 |
| Liver cancer | 4 |
| Bladder cancer | 3 |
| Breast cancer | 3 |
| Leukaemia | 3 |
| Melanoma | 2 |
| Rectal cancer | 2 |
| Thyroid cancer | 2 |
| Anal cancer | 1 |
| Cancer of heart, mediastinum and pleura | 1 |
| Connective tissue cancer (Sarcoma) | 1 |
| Oesophageal cancer | 1 |
| Oral cavity cancer | 1 |
| Ovarian cancer | 1 |
| Stomach cancer | 1 |
| Testicular cancer | 1 |
| Tonsil cancer | 1 |
| Unknown | 1 |

# Supplementary Table 2. Plasma levels of immune regulation and senescence-associated secretory phenotype (SASP) biomarkers at baseline in people with HIV (PWH) on antiretroviral treatment without and with non–AIDS–defining cancers (NADCs) during the follow–up time.

|  |  | **NADCs (Median and 95%CI)** | | | **Unadjusted GLM** | | **Adjusted GLM** | |
| --- | --- | --- | --- | --- | --- | --- | --- | --- |
| **Category** | **Marker** | **Yes** | **No** | ***p-value*** | **AMR (95%CI)** | ***p-value*** | **aAMR (95%CI)** | ***p-value*** |
| Immune  regulation | CD28 | 19.23 (13.63 – 24.64) | 15.32 (13.45 – 19.95) | 0.475 | 0.97 (0.65 – 1.44) | 0.864 | 1.10 (0.70 – 1.72) | 0.679 |
|  | CD80 | 11.95 (10.33 – 12.77) | 11.47 (10.85 – 11.95) | 0.902 | 0.95 (0.84 – 1.08) | 0.461 | 0.96 (0.84 – 1.11) | 0.597 |
|  | PD–1 | 44.08 (34.45 – 77.18) | 40.70 (37.06 – 48.12) | 0.591 | 1.09 (0.72 – 1.65) | 0.667 | 1.11 (0.71 – 1.74) | 0.672 |
|  | PD–L1 | 15.56 (12.82 – 18.86) | 14.79 (13.91 – 16.04) | 0.696 | 1.11 (0.84 – 1.47) | 0.473 | 1.11 (0.81 – 1.51) | 0.540 |
|  | PD–L2 | 24.19 (22.13 – 27.76) | 22.17 (22.12 – 24.19) | 0.222 | 1.04 (0.91 – 1.19) | 0.542 | 1.07 (0.93 – 1.24) | 0.351 |
|  | LAG–3 | 12.75 (11.16 – 15.03) | 12.35 (11.35 – 13.71) | 0.411 | 1.02 (0.70 – 1.49) | 0.918 | 1.01 (0.67 – 1.51) | 0.973 |
|  | TIM–3 | 108.60 (96.15 – 132.67) | 98.14 (91.84 – 107.12) | 0.291 | 1.10 (0.89 – 1.36) | 0.387 | 1.08 (0.85 – 1.36) | 0.548 |
| SASP | CD36 | 13.74 (10.88 – 16.05) | 12.54 (11.44 – 13.54) | 0.216 | 1.25 (0.94 – 1.68) | 0.121 | 1.25 (0.91 – 1.71) | 0.175 |
|  | IL–1α | 35.09 (25.31 – 42.36) | 32.82 (28.37 – 37.02) | 0.722 | 1.08 (0.84 – 1.39) | 0.545 | 1.09 (0.82 – 1.43) | 0.570 |
|  | IL–8 | 47.96 (36.18 – 67.85) | 51.06 (42.60 – 62.83) | 0.945 | 0.93 (0.70 – 1.23) | 0.614 | 1.03 (0.76 – 1.41) | 0.846 |
|  | MCP–1 | 16.78 (13.43 – 21.71) | 17.00 (16.65 – 18.39) | 0.780 | 0.93 (0.75 – 1.14) | 0.491 | 0.90 (0.72 – 1.13) | 0.384 |
|  | MCP–2 | 18.69 (16.49 – 22.52) | 18.69 (17.56 – 20.01) | 0.599 | 0.88 (0.67 – 1.17) | 0.396 | 0.82 (0.61 – 1.10) | 0.209 |
|  | TNF–β | 81.37 (59.57 – 103.49) | 86.53 (73.67 – 114.77) | 0.310 | 0.80 (0.63 – 1.02) | 0.081 | 0.85 (0.65 – 1.11) | 0.239 |
|  | TNF–RI | 101.68 (57.96 – 148.32) | 80.12 (64.05 – 95.31) | 0.414 | 1.08 (0.79 – 1.48) | 0.640 | 1.03 (0.72 – 1.47) | 0.883 |
|  | TNF–RII | 117.92 (102.19 – 136.52) | 107.89 (101.86 – 118.30) | 0.223 | 1.08 (0.92 – 1.28) | 0.344 | 1.01 (0.84 – 1.21) | 0.958 |
|  | IP–10 | 47.79 (39.06 – 63.44) | 43.60 (37.69 – 57.55) | 0.869 | 0.95 (0.69 – 1.33) | 0.777 | 1.06 (0.74 – 1.53) | 0.759 |
|  | SDF–1α | 42.93 (33.10 – 50.76) | 39.69 (36.18 – 44.57) | 0.549 | 1.10 (0.89 – 1.35) | 0.389 | 1.07 (0.85 – 1.34) | 0.567 |
|  | GDF–15 | 84.22 (59.55 – 105.07) | 67.54 (59.55 – 77.55) | 0.205 | 1.08 (0.82 – 1.44) | 0.580 | 1.10 (0.81 – 1.51) | 0.539 |
|  | PAI–1 | 19.57 (17.02 – 21.79) | 17.17 (16.05 – 18.86) | 0.071 | 1.04 (0.93 – 1.17) | 0.482 | 1.05 (0.93 – 1.20) | 0.425 |
|  | EGF | 15.24 (11.21 – 20.39) | 14.55 (13.49 – 16.75) | 0.703 | 1.14 (0.78 – 1.65) | 0.490 | 1.19 (0.78 – 1.81) | 0.432 |
|  | HGF | 11.20 (10.32 – 12.27) | 10.09 (9.42 – 10.53) | 0.085 | 1.01 (0.83 – 1.23) | 0.938 | 1.06 (0.85 – 1.32) | 0.618 |
|  | FGF–2 | 33.85 (25.97 – 53.31) | 31.99 (25.97 – 35.39) | 0.362 | 1.14 (0.77 – 1.70) | 0.510 | 1.21 (0.78 – 1.87) | 0.415 |
|  | VEGF–A | 72.70 (64.27 – 87.50) | 73.56 (64.27 – 81.13) | 0.544 | 1.08 (0.87 – 1.34) | 0.472 | 1.07 (0.85 – 1.36) | 0.554 |
|  | MMP–1 | 10.34 (8.73 – 10.95) | 9.48 (8.75 – 10.08) | 0.574 | 1.03 (0.87 – 1.21) | 0.760 | 1.07 (0.89 – 1.28) | 0.476 |

**Statistics**: Medians are presented as arbitrary units (a.u.). P-values for the comparison between groups were calculated using the Mann-Whitney U test. AMR were calculated by Generalized Linear Models (GLM) with a gamma distribution (log-link). Multivariable models were adjusted for relevant confounders. **Abbreviations**: AMR, arithmetic mean ratio; aAMR, adjusted AMR; 95%CI, 95% of confidence interval; p, level of significance; CD, cluster of differentiation; EGF, epidermal growth factor; FGF, fibroblast growth factor; GDF, growth differentiation factor; HGF, hepatocyte growth factor; IL, interleukin; IP, interferon gamma–induced protein; LAG, lymphocyte activation gene; MCP, monocyte chemoattractant protein; MMP, matrix metalloproteinase; PAI, Plasminogen Activator Inhibitor; PD–1, programmed cell death protein; PD–L, programmed death–ligand; SDF, stromal cell–derived factor ; TIM, T–cell immunoglobulin and mucin domain; TNF, tumour necrosis factor; TNF–R, tumour necrosis factor receptor; VEGF, vascular endothelial growth factor.

# Supplementary Table 3. Association of plasma markers of immune regulation and senescence-associated secretory phenotype (SASP) at baseline with the development of non–AIDS–defining cancers (NADCs) during the follow–up time in people with HIV (PWH) on antiretroviral treatment.

|  |  | **Unadjusted** | | | **Adjusted** | | |
| --- | --- | --- | --- | --- | --- | --- | --- |
| **Category** | **Marker** | **HR (95%CI)** | ***p-value*** | ***q-value*** | **aHR (95%CI)** | ***p-value*** | ***q-value*** |
| Immune regulation | CD28 | 1.26 (1.01 – 1.56) | **0.036** | 0.217 | 1.31 (1.03 – 1.66) | **0.029** | **0.070** |
|  | CD80 | 1.30 (0.75 – 2.26) | 0.343 | 0.549 | 1.41 (0.70 – 2.83) | 0.341 | 0.390 |
|  | PD–1 | 1.15 (0.95 – 1.41) | 0.157 | 0.367 | 1.15 (0.93 – 1.42) | 0.191 | 0.250 |
|  | PD–L1 | 1.27 (0.95 – 1.70) | 0.112 | 0.367 | 1.55 (1.16 – 2.07) | **0.003** | **0.016** |
|  | PD–L2 | 2.04 (1.27 – 3.26) | **0.003** | **0.072** | 3.33 (1.92 – 5.80) | **<0.001** | **<0.001** |
|  | LAG–3 | 1.13 (0.89 – 1.45) | 0.317 | 0.549 | 1.31 (1.01 – 1.70) | **0.039** | **0.081** |
|  | TIM–3 | 1.31 (0.90 – 1.89) | 0.157 | 0.367 | 1.56 (1.05 – 2.30) | **0.027** | **0.070** |
| Senescence–associated secretory phenotype (SASP) | CD36 | 1.28 (0.96 – 1.70) | 0.089 | 0.358 | 1.56 (1.11 – 2.19) | **0.010** | **0.034** |
|  | IL–1α | 1.09 (0.79 – 1.50) | 0.608 | 0.708 | 1.43 (1.02 – 2.01) | **0.041** | **0.081** |
|  | IL–8 | 1.20 (0.91 – 1.58) | 0.197 | 0.395 | 1.33 (0.98 – 1.80) | 0.066 | 0.111 |
|  | MCP–1 | 1.12 (0.75 – 1.67) | 0.576 | 0.708 | 1.30 (0.84 – 2.00) | 0.235 | 0.281 |
|  | MCP–2 | 1.08 (0.79 – 1.49) | 0.620 | 0.708 | 1.27 (0.88 – 1.82) | 0.198 | 0.250 |
|  | TNF–β | 0.92 (0.70 – 1.22) | 0.581 | 0.708 | 0.97 (0.67 – 1.39) | 0.857 | 0.858 |
|  | TNF–RI | 1.02 (0.80 – 1.30) | 0.860 | 0.938 | 1.08 (0.85 – 1.38) | 0.520 | 0.567 |
|  | TNF–RII | 0.89 (0.58 – 1.37) | 0.603 | 0.708 | 1.44 (0.86 – 2.40) | 0.169 | 0.238 |
|  | IP–10 | 1.01 (0.76 – 1.34) | 0.945 | 0.945 | 1.03 (0.74 – 1.45) | 0.858 | 0.858 |
|  | SDF–1α | 0.90 (0.62 – 1.31) | 0.591 | 0.708 | 1.48 (0.99 – 2.23) | 0.058 | 0.107 |
|  | GDF–15 | 1.43 (1.08 – 1.90) | **0.013** | **0.106** | 1.47 (1.09 – 1.99) | **0.013** | **0.038** |
|  | PAI–1 | 1.49 (0.89 – 2.51) | 0.128 | 0.367 | 2.27 (1.23 – 4.19) | **0.009** | **0.034** |
|  | EGF | 1.13 (0.88 – 1.46) | 0.336 | 0.549 | 1.26 (0.98 – 1.62) | 0.069 | 0.111 |
|  | HGF | 1.63 (1.14 – 2.34) | **0.008** | **0.090** | 1.88 (1.32 – 2.68) | **<0.001** | **0.006** |
|  | FGF–2 | 1.26 (0.99 – 1.60) | 0.062 | 0.298 | 1.45 (1.10 – 1.91) | **0.008** | **0.034** |
|  | VEGF–A | 1.02 (0.68 – 1.53) | 0.912 | 0.945 | 1.50 (0.94 – 2.40) | 0.092 | 0.138 |
|  | MMP–1 | 1.41 (0.86 – 2.31) | 0.168 | 0.367 | 2.32 (1.40 – 3.84) | **0.001** | **0.008** |

**Statistics**: HR and 95%CI were estimated with cause–specific Cox proportional hazards regression models with Borgan II weights. with age as time scale. Multivariable models were adjusted for relevant confounders (see Methods Section). The q–values represent p–values corrected for multiple testing using the False Discovery Rate (FDR). Statistically significant differences are shown in bold. **Abbreviations**: HR, Hazard ratio; aHR, adjusted HR; 95%CI, 95% of confidence interval; p, level of significance; q, corrected level of significance; CD, cluster of differentiation; EGF, epidermal growth factor; FGF, fibroblast growth factor; GDF, growth differentiation factor; HGF, hepatocyte growth factor; IL, interleukin; IP, interferon gamma–induced protein; LAG, lymphocyte activation gene; MCP, monocyte chemoattractant protein; MMP, matrix metalloproteinase; PAI, Plasminogen Activator Inhibitor; PD–1, programmed cell death protein; PD–L, programmed death–ligand; SDF, stromal cell–derived factor ; TIM, T–cell immunoglobulin and mucin domain; TNF, tumour necrosis factor; TNF–R, tumour necrosis factor receptor; VEGF, vascular endothelial growth factor.

# Supplementary Table 4. Plasma levels of immune regulation and senescence-associated secretory phenotype (SASP) biomarkers at baseline in people with HIV (PWH) on antiretroviral treatment stratified by gender and NADC development.

| **Category** | **Marker** | **NADCs** | | | | | |
| --- | --- | --- | --- | --- | --- | --- | --- |
|  |  | **Yes** | | | **No** | | |
|  |  | **Male** | **Female** | ***p-value*** | **Male** | **Female** | ***p-value*** |
| Immune  regulation | CD28 | 18.17 (13.63 – 30.65) | 21.28 (9.08 – 29.79) | 0.426 | 15.66 (13.63 – 20.82) | 12.48 (10.25 – 26.23) | 0.797 |
|  | CD80 | 11.95 (10.85 – 13.83) | 10.89 (7.26 – 13.15) | 0.114 | 11.57 (10.85 – 11.95) | 10.85 (9.77 – 12.90) | 0.567 |
|  | PD–1 | 44.08 (31.08 – 77.18) | 44.62 (18.61 – 219.53) | 0.783 | 42.79 (38.13 – 48.42) | 39.12 (26.23 – 58.86) | 0.699 |
|  | PD–L1 | 16.10 (12.82 – 18.87) | 15.24 (10.41 – 20.88) | 0.972 | 15.15 (14.14 – 16.34) | 13.83 (9.80 – 15.31) | 0.066 |
|  | PD–L2 | 23.92 (22.12 – 25.40) | 27.67 (20.21 – 36.45) | 0.499 | 22.44 (22.12 – 24.90) | 22.17 (17.66 – 28.54) | 0.842 |
|  | LAG–3 | 12.75 (11.16 – 15.03) | 12.42 (9.49 – 22.98) | 0.893 | 12.43 (11.60 – 14.64) | 10.32 (8.17 – 14.64) | 0.055 |
|  | TIM–3 | 103.85 (91.38 – 132.67) | 124.99 (68.09 – 223.88) | 0.472 | 100.47 (92.32 – 112.02) | 88.47 (68.81 – 108.16) | 0.216 |
| SASP | CD36 | 13.75 (10.94 – 20.44) | 11.44 (8.90 – 16.00) | 0.153 | 12.54 (11.44 – 13.74) | 12.36 (10.12 – 14.42) | 0.685 |
|  | IL–1α | 38.78 (26.97 – 45.91) | 27.45 (20.30 – 42.36) | 0.327 | 32.82 (28.37 – 37.19) | 30.50 (22.16 – 48.39) | 0.515 |
|  | IL–8 | 49.15 (37.25 – 91.32) | 42.66 (33.27 – 56.91) | 0.499 | 54.87 (42.60 – 67.30) | 43.61 (29.19 – 54.00) | 0.292 |
|  | MCP–1 | 16.10 (12.70 – 21.30) | 21.27 (11.34 – 37.27) | 0.410 | 17.93 (16.78 – 18.95) | 13.43 (11.71 – 17.93) | **0.008** |
|  | MCP–2 | 18.69 (16.49 – 23.50) | 18.69 (13.89 – 27.36) | 0.844 | 18.69 (17.56 – 20.14) | 18.33 (12.52 – 21.38) | 0.473 |
|  | TNF–β | 81.83 (55.32 – 113.48) | 81.37 (58.40 – 153.61) | 0.767 | 87.38 (73.91 – 115.75) | 83.83 (45.39 – 128.83) | 0.392 |
|  | TNF–RI | 102.27 (57.50 – 140.78) | 87.33 (55.47 – 267.14) | 0.459 | 85.80 (71.16 – 100.80) | 62.55 (49.77 – 138.50) | 0.605 |
|  | TNF–RII | 117.08 (102.19 – 134.01) | 120.88 (90.06 – 161.89) | 0.568 | 108.28 (102.19 – 119.78) | 102.35 (67.85 – 127.38) | 0.315 |
|  | IP–10 | 50.62 (40.00 – 65.62) | 42.30 (19.29 – 60.79) | 0.197 | 43.47 (37.69 – 58.03) | 47.38 (24.99 – 74.08) | 0.676 |
|  | SDF–1α | 43.76 (37.46 – 50.76) | 31.01 (27.19 – 57.58) | 0.627 | 40.27 (36.18 – 44.93) | 37.18 (29.68 – 49.35) | 0.508 |
|  | GDF–15 | 84.77 (59.55 – 117.76) | 70.88 (48.83 – 129.83) | 0.693 | 69.22 (60.37 – 80.35) | 51.04 (38.15 – 87.92) | 0.114 |
|  | PAI–1 | 18.29 (16.20 – 21.44) | 21.44 (17.25 – 29.57) | 0.153 | 17.04 (15.76 – 18.86) | 17.17 (15.78 – 20.60) | 0.565 |
|  | EGF | 16.75 (11.21 – 21.81) | 13.81 (9.70 – 20.39) | 0.494 | 14.90 (13.60 – 16.98) | 11.21 (9.56 – 19.16) | 0.295 |
|  | HGF | 11.20 (9.42 – 12.27) | 11.20 (10.32 – 15.16) | 0.352 | 10.44 (9.42 – 11.20) | 8.82 (7.92 – 9.42) | **0.032** |
|  | FGF–2 | 35.99 (25.97 – 54.84) | 33.85 (20.86 – 67.81) | 0.983 | 32.06 (27.01 – 35.89) | 22.44 (16.48 – 37.96) | 0.101 |
|  | VEGF–A | 75.12 (65.24 – 93.17) | 62.60 (42.99 – 120.68) | 0.508 | 74.58 (63.77 – 82.04) | 67.06 (48.57 – 91.37) | 0.306 |
|  | MMP–1 | 10.58 (8.73 – 10.95) | 8.94 (7.01 – 15.20) | 0.632 | 9.56 (8.95 – 10.34) | 8.60 (7.20 – 9.54) | 0.065 |

**Statistics**: Data are presented as medians (arbitrary units, a.u.) and 95%CI. P-values for the comparison between groups were calculated using the Mann-Whitney U test to compare biomarker levels between males and females within each group (NADC cases and non-NADC subcohort).. **Abbreviations**: 95%CI, 95% of confidence interval; p, level of significance; CD, cluster of differentiation; EGF, epidermal growth factor; FGF, fibroblast growth factor; GDF, growth differentiation factor; HGF, hepatocyte growth factor; IL, interleukin; IP, interferon gamma–induced protein; LAG, lymphocyte activation gene; MCP, monocyte chemoattractant protein; MMP, matrix metalloproteinase; PAI, Plasminogen Activator Inhibitor; PD–1, programmed cell death protein; PD–L, programmed death–ligand; SDF, stromal cell–derived factor ; TIM, T–cell immunoglobulin and mucin domain; TNF, tumour necrosis factor; TNF–R, tumour necrosis factor receptor; VEGF, vascular endothelial growth factor.

# Supplementary Table 5. Unadjusted association of plasma markers of immune regulation and senescence-associated secretory phenotype (SASP) at baseline with the development of non–AIDS–defining cancers (NADCs) during the follow–up time in people with HIV (PWH) on antiretroviral treatment, by gender.

|  |  | **Female individuals** | **Male individuals** | **Interaction** | |
| --- | --- | --- | --- | --- | --- |
| **Category** | **Marker** | **HR (95%CI)** | **HR (95%CI)** | ***p-value*** | ***q-value*** |
| Immune regulation | CD28 | 1.06 (0.48 – 2.31) | 1.29 (1.03 – 1.61) | 0.478 | 0.604 |
|  | CD80 | 0.24 (0.02 – 2.88) | 1.54 (0.89 – 2.66) | **0.021** | **0.085** |
|  | PD–1 | 1.35 (0.75 – 2.43) | 1.12 (0.91 – 1.37) | 0.358 | 0.477 |
|  | PD–L1 | 1.97 (1.18 – 3.27) | 1.16 (0.83 – 1.63) | 0.080 | 0.240 |
|  | PD–L2 | 3.59 (1.12 – 11.53) | 1.84 (1.10 – 3.08) | 0.183 | 0.367 |
|  | LAG–3 | 1.54 (0.95 – 2.49) | 1.06 (0.8 – 1.40) | 0.149 | 0.324 |
|  | TIM–3 | 2.38 (1.04 – 5.45) | 1.16 (0.76 – 1.75) | 0.058 | 0.199 |
| Senescence–associated secretory phenotype (SASP) | CD36 | 1.26 (0.61 – 2.61) | 1.29 (0.96 – 1.74) | 0.941 | 0.982 |
|  | IL–1α | 1.37 (0.46 – 4.02) | 1.07 (0.77 – 1.48) | 0.549 | 0.627 |
|  | IL–8 | 1.01 (0.44 – 2.36) | 1.23 (0.92 – 1.65) | 0.531 | 0.627 |
|  | MCP–1 | 2.73 (1.40 – 5.35) | 0.89 (0.56 – 1.42) | **0.002** | **0.022** |
|  | MCP–2 | 1.82 (0.73 – 4.58) | 1.01 (0.72 – 1.42) | 0.140 | 0.324 |
|  | TNF–β | 0.69 (0.34 – 1.40) | 0.98 (0.73 – 1.32) | 0.219 | 0.397 |
|  | TNF–RI | 1.25 (0.61 – 2.58) | 0.98 (0.77 – 1.26) | 0.343 | 0.477 |
|  | TNF–RII | 2.63 (1.13 – 6.14) | 0.76 (0.48 – 1.20) | **0.005** | **0.031** |
|  | IP–10 | 0.54 (0.27 – 1.09) | 1.19 (0.90 – 1.58) | **0.001** | **0.022** |
|  | SDF–1α | 1.30 (0.63 – 2.69) | 0.85 (0.56 – 1.28) | 0.232 | 0.397 |
|  | GDF–15 | 1.51 (0.74 – 3.06) | 1.42 (1.05 – 1.93) | 0.849 | 0.926 |
|  | PAI–1 | 3.21 (0.77 – 13.28) | 1.30 (0.75 – 2.25) | 0.147 | 0.324 |
|  | EGF | 1.38 (0.77 – 2.49) | 1.09 (0.83 – 1.43) | 0.320 | 0.477 |
|  | HGF | 4.00 (1.89 – 8.47) | 1.38 (0.93 – 2.05) | **0.005** | **0.031** |
|  | FGF–2 | 1.52 (0.99 – 2.30) | 1.20 (0.92 – 1.58) | 0.305 | 0.477 |
|  | VEGF–A | 1.02 (0.23 – 4.61) | 1.03 (0.68 – 1.55) | 0.990 | 0.990 |
|  | MMP–1 | 3.81 (1.34 – 10.88) | 1.23 (0.72 – 2.10) | **0.021** | **0.085** |

**Statistics**: Data were calculated by Cox proportional hazards regression models with Borgan weighting. Death was used as a competing event in the models. The q–values represent p–values corrected for multiple testing using the False Discovery Rate (FDR). Statistically significant differences are shown in bold. **Abbreviations**: HR, Hazard Ratio; 95%CI, 95% of confidence interval; p, level of significance; q, corrected level of significance; CD, cluster of differentiation; EGF, epidermal growth factor; FGF, fibroblast growth factor; GDF, growth differentiation factor; HGF, hepatocyte growth factor; IL, interleukin; IP, interferon gamma–induced protein; LAG, lymphocyte activation gene; MCP, monocyte chemoattractant protein; MMP, matrix metalloproteinase; PAI, Plasminogen Activator Inhibitor; PD–1, programmed cell death protein; PD–L, programmed death–ligand; SDF, stromal cell–derived factor ; TIM, T–cell immunoglobulin and mucin domain; TNF, tumour necrosis factor; TNF–R, tumour necrosis factor receptor; VEGF, vascular endothelial growth factor.

# Supplementary Figure 1. Correlation matrix of plasma levels of immune regulation and senescence-associated secretory phenotype (SASP) biomarkers at baseline in people with HIV (PWH) on antiretroviral treatment.


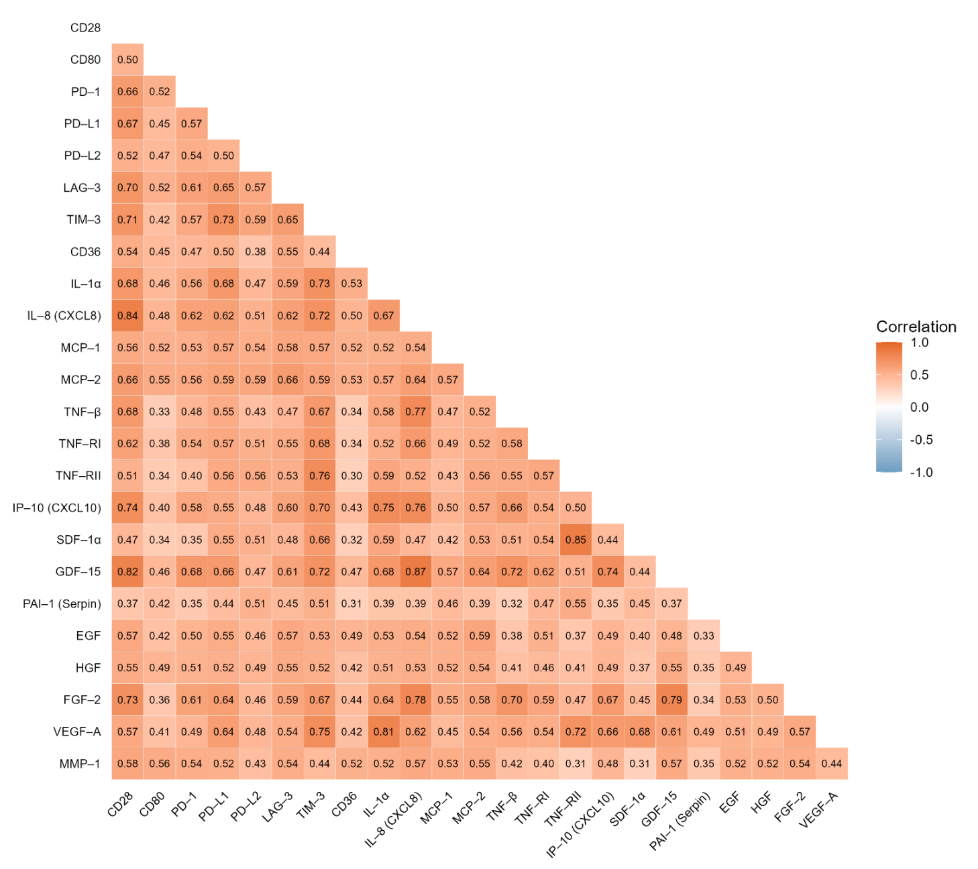


Heatmap of pairwise Spearman's rank correlation coefficients between the 24 measured biomarkers at baseline. Each cell contains the Spearman's correlation coefficient (ρ). The color scale represents the correlation strength, where darker orange indicates a stronger positive correlation. The numerical values within each cell represent the specific correlation coefficient. All pairwise correlations shown were highly statistically significant (p < 0.001).
